# Understanding Formative Assessment Practice in the EFL Exam-Oriented Context: An Application of the Theory of Planned Behavior

**DOI:** 10.3389/fpsyg.2021.774159

**Published:** 2021-12-09

**Authors:** Juan Zeng, Liyan Huang

**Affiliations:** The Center for Language Cognition and Assessment, School of Foreign Studies, South China Normal University, Guangzhou, China

**Keywords:** formative assessment, EFL, theory of planned behavior, Chinese context, exam-oriented

## Abstract

Formative assessment (FA) has been used to facilitate EFL learning and teaching. However, due to factors such as task complexity and time constraints, FA implementation faces a variety of challenges, especially in countries with an exam-oriented education system. Drawing on the case of EFL teachers from Chinese public secondary schools, this study examines the features of FA practice and explains their underlying aspects in an EFL exam-oriented context from a social psychology perspective. It adopts a mixed-methods research approach. Guided by the theory of planned behavior, 10 English teachers from Guangdong province in China were interviewed to establish an item pool for a structured questionnaire. A total of 161 English teachers from four cities in Guangdong province took part in the subsequent survey. The results revealed that the participating teachers have an implicit understanding of FA, based primarily on its literal meaning and their own teaching experience. They know and follow FA methods but lack confidence about their own practice. Regional differences were significant. Possible reasons for the perceptions and practices of Chinese EFL teachers from public secondary schools are the teachers’ own attitudes, the influence of other stakeholders, and the limitations of the FA methods. The study elucidates the features of FA practice and its mechanism in an EFL exam-oriented context.

## Introduction

As an effective learning-improvement strategy and a useful teaching-aid, formative assessment (FA) has recently received growing attention in the EFL classroom. Initiatives in educational policy reform attempting to facilitate FA to bring about changes in teaching and learning have been taken throughout the world, for instance, in the United States (Bunch, 2011), Hong Kong (Davison, 2004), and in Europe (Jones, 2009). In China, educational policy reform has also witnessed a shift from the general use of summative assessment (SA) to the promotion of FA [Ministry of Education of the People’s Republic of China (MoE), 2011]. In secondary education, which is traditionally exam-oriented, the national matriculation test (*Gaokao*) and the high school entrance examinations (Z*hongkao*) have been regarded as the primary purpose of teaching practice. “Teaching to the test” is common in the EFL classroom. The new national English curriculum standards highlighted the role of FA in EFL practice. Teachers are being encouraged to improve the effectiveness of their instruction by integrating teaching and assessment [Ministry of Education of the People’s Republic of China (MoE), 2017]. Significant efforts have been made to improve EFL teachers’ understanding of FA and to facilitate the implementation of assessment tasks. However, for decades, English teachers in Chinese secondary schools have been obsessed with large-scale high-stakes testing. In this context, teaching has been teacher-centered, test-centered, and textbook-centered (Cheng, 2010). Although FA has been advocated by stakeholders such as experts and teacher educators, and its benefits have been acknowledged, it has not been fully adopted in classrooms (e.g., Wang, 2017). According to the literature, the benefits of integrating FA into the EFL classroom lie in the significant effect on improving students’ learning and self-regulation skills (Black and Wiliam, 1998; Hattie and Timperley, 2007). Specifically, students can be made aware of their learning strengths and weaknesses through the identification of the gaps that exist between their learning goal and their current knowledge and skills. In view of the historical background, how teachers actually perceive FA and how they adapt FA methods to a real classroom need to be explored.

Studies have shown that effective FA methods can serve as a teaching and learning tool to improve students’ achievements (Black and Wiliam, 1998) and to raise the quality of the educational system (Sadler, 1989). Theoretical studies have discussed FA principles (Wiliam, 2011), theoretical frameworks (Stiggins, 1992), and assessment methods (Andrade and Du, 2007). Numerous empirical studies have explored its effects in language skills development and in the learning of specific subjects (Cumming, 2001; Li, 2010). Although FA can improve students’ achievement, and it has been advocated by policy-makers (Wu et al., 2021) because it can foster students’ learning autonomy [Ministry of Education of the People’s Republic of China (MoE), 2017], FA approaches are not widely used in daily teaching practice, especially in the exam-oriented context.

Previous research has shown that EFL teachers struggle to implement FA methods into classroom practice (e.g., Wang, 2017). While researchers identified that factors such as teachers’ FA competence and the time at their disposal to employ FA practice could have a negative influence on FA implementation (Volante and Beckett, 2011; Smit and Birri, 2014), few studies have systematically investigated the factors that are essential for successful FA from the social psychology perspective. In the EFL classroom, the implementation of FA strategies is a complicated social behavior involving many factors such as teachers’ beliefs, students’ perceptions, institutional differences, education policy, and the examination system (Cowan, 2009; Carless, 2011; Chen et al., 2014). The educational context has been identified as a potentially crucial factor influencing the translation of FA theories into practice, especially for Chinese EFL teachers (Brown et al., 2011). In order to systematically examine and explain the mechanism of FA implementation, the present study employs the theory of planned behavior (TPB) as proposed by Ajzen (1985). TPB offers a theoretical framework to categorize and assess various factors from the social psychology perspective. The influences uncovered in the research literature can be accommodated within the framework. In an exam-dominated educational context, it is still unknown how well FA works in Chinese secondary schools (i.e., junior and senior high schools), and there is a need to identify the factors determining the current state of FA implementation. Therefore, the present study aims to apply Ajzen’s (1985) TPB to investigate EFL teachers’ perceptions and practice of FA in Chinese secondary schools and to identify the factors that may facilitate or impede FA implementation.

## Formative Assessment and the Theory of Planned Behavior

Formative assessment is interpreted as “encompassing all those activities undertaken by teachers, and/or by their students, which provide information to be used as feedback to modify the teaching and learning activities in which they are engaged” (Black and Wiliam, 1998, pp. 7–8). It is an ongoing process of gathering evidence by different methods, such as feedback and questioning. (Black et al., 2004; Heritage, 2007). These methods can provide teachers with evidence to support making adjustments to teaching and learning (Airasian, 2001). The main aspects of participants’ involvement in FA have been identified as relating to teachers, peers, and the learners themselves (Wiliam and Thompson, 2008). In addition, Volante and Beckett’s (2010) investigation reported that teachers also consider the role of parents. Some studies have investigated students’ perceptions of FA. Cheng et al. (2015) explored Chinese university students’ perceptions of assessment tasks, revealing that students’ perceptions are closely related to the classroom assessment environment. Regarding teachers’ perceptions, studies show that Chinese EFL teachers have an accurate understanding of FA while persisting in the use of the summative interpretation of written rating scales (Xue and Tang, 2012). The conflict between teachers’ perceptions and practices arises from their perceptions and habits regarding grading.

Various FA methods have been suggested in the relevant literature. In the educational context in China, methods such as providing feedback, questioning, portfolio assessment, self-assessment, peer assessment, and formative use of summative tests are commonly used by Chinese EFL teachers (Wang, 2017). The practice of FA is complex. Previous studies investigated students’ beliefs regarding FA methods, such as peer assessment (Ghahari and Sedaghat, 2018) and feedback (Xiao and Yang, 2019). Findings from some studies reported students’ sound understanding of these methods, while others showed that students had an underdeveloped conception of FA (e.g., MacLellan, 2001). These inconsistent findings indicate that the teaching context and the teachers’ instructions are of great importance. The methods used during FA activities also matter. Kepner (1991) found that message-related feedback is more useful than surface error corrections in helping students avoid making errors in L2 writing.

The actual effects of FA methods are varied for other reasons. Hattie and Timperley (2007) measured the effects of feedback with a large effect size of 0.73. However, due to teachers’ misunderstanding and misuse of feedback, Lee’s (2007) study did not show that the implementation of feedback brought about any improvement in students’ L2 writing. As Black et al. (2004, p. 13) found, questions are useful for raising issues for which a teacher needs information or which the students need to consider. Frameworks for framing quality questions, such as Anderson and Krathwohl’s (2001) taxonomy, can be effective if adopted wisely. However, the actual practice is often not as effective as claimed because teachers do not give students enough time to respond (Black and Wiliam, 2010). The probable reason is that teachers are under time pressure to get through the teaching content. There is a general agreement on the value of portfolio assessment in improving students’ written language accuracy and coherence (Li, 2010). However, the time and energy devoted to portfolio assessment are substantial, leading to the development of electronic portfolios (Yastibas and Yastibas, 2015). Studies have provided convincing evidence that self-assessment plays a fundamental role in providing an opportunity for students to monitor their learning process (Wiliam, 2011). Peers also provide constructive feedback on students’ performance, especially in a large class (Ballantyne et al., 2002). For example, collaborative writing with peer assessment can improve writing quality (Yarrow and Topping, 2001). However, when Klimenko and Sleptsova (2015) applied the same strategy to a course in “English Speech Practice,” they identified students’ underdeveloped ability to assess peers’ work. Teachers’ FA practice in the Chinese context is sophisticated. Chinese EFL teachers show a high level of synergy between FA and SA (Wang, 2017). The formative use of summative tests aims to use the information generated in tests to recognize high school students’ learning needs and to help to improve their understanding of their test performance (Xiao, 2017). However, when teachers rely on tests to understand their students’ learning performance (Harlen, 2006), there is a temptation for them to teach to improve their students’ test results.

Apart from research done to investigate the efficiency of FA, emphasis has been placed on exploring the factors that influence FA practice. Carless (2011) argued that the influencing factors covered three strands: the teacher, the school, and the educational context. Teachers’ adoption of FA principles and practices could facilitate FA practice. In line with this factor, Lyon et al.’s (2021) case study found that teachers with low content knowledge tended to implement the less advanced aspects of FA, indicating that the relevant professional support was lacking. School factors such as school leaders’ support are indispensable because they will ensure the availability of resources. In the educational context, compatibility between government policies, curriculum goals, the examination system, and FA ideals have a broad impact. Furthermore, Lyon et al.’s (2021) study confirmed the existence of weaknesses in the application of FA strategies. Although it has been shown that certain factors influence FA implementation, and researchers have been aware that assessment practice in EFL teaching is a social behavior, scant attention has been paid to how these factors work systematically to facilitate or inhibit FA implementation from a social psychological perspective.

As demonstrated above, the reasons for the unexpected effects of actual FA methods differ. The explanation of this phenomenon uses the TPB, one of the most widely used models employed to explain human behavior from a social psychological perspective (Ajzen, 1985, 1991). According to the TPB, people’s intention to engage in a particular behavior is guided by three factors: attitudes (favorable or unfavorable views), subjective norms (the pressure or influence from social relationships), and perceived behavioral control (the judgment of the extent to which a behavior is within the person’s control). In short, it is suggested that teachers’ understanding of FA, the capacity of teachers and students to implement FA, together with the negative impact of some FA methods, could explain teachers’ decision-making behavior concerning FA practice.

Previous studies have discussed the efficacy of FA practice, but the findings are inconsistent. As demonstrated in earlier studies, attempts made by Chinese EFL teachers to adapt FA to classroom practice are significantly influenced by the prevailing exam-oriented context. This context involves a complex interplay between FA and SA practices (Carless, 2011; Xiao, 2017). The extent to which FA has been implemented in the context of Chinese secondary education, with its highly competitive exam-oriented educational system, has rarely been addressed. To date, issues relating to Chinese EFL teachers’ use of FA in public secondary schools, at least on a large scale, have scarcely been investigated. Therefore, this study attempts to present a holistic picture of the current situation of FA implementation in a high-stakes testing context. Furthermore, because little is known about teachers’ decision-making concerning FA, the factors underlying the current situation are explored. The study was guided by the following three research questions:

RQ1: What are the perceptions of formative assessment of English teachers in secondary schools in China?

RQ2: To what extent are formative assessment methods used in English instruction in secondary schools in China?

RQ3: What factors might account for the implementation of formative assessment in secondary schools in China?

## Research Methodology

The study adopted an exploratory sequential mixed-method approach to answer the research questions. Initially, qualitative data were collected, and a phase of quantitative data collection followed. This process enabled a comprehensive understanding of the phenomena (Creswell and Plano Clark, 2011; Cohen et al., 2018). Within the design, multiple sources of data were sampled on the same issue to gain a thorough understanding of the reality of FA practice in Chinese public secondary schools and to explore the underlying factors that explain the situation. Specifically, 10 teachers were interviewed. The interview data were analyzed qualitatively to initially grasp their perceptions and practice of FA in an EFL context. In the quantitative phase, valid data were obtained from 157 questionnaires to generalize the findings collected in the qualitative phase.

### Context of the Study

The study targeted EFL teachers from public secondary schools, and it was conducted in Guangdong province in Southern China. The secondary education system in China includes junior high and senior high schools, the majority of which are state-run and only a few private schools. Young people receive nine years of compulsory education, six years in primary school, followed by three years of junior high school. After their compulsory education, young people may sit the *Zhongkao*, the high school entrance examination, to enter senior high school. Not all students who sit the exam succeed in passing. Those who do can choose a senior high school according to their performance. After three further years of learning, they may take the *Gaokao*, the national matriculation test, which determines whether they can enter university for further study. These two high-stakes national examinations, *Zhongkao* and *Gaokao*, are of great importance to students’ futures. In that context, teachers and students experience substantial test pressure (Cheng and Qi, 2006; Xiao, 2017).

Considering the extent to which they represent the development of the economy and education in Guangdong province, the study targeted Guangzhou (A) and Foshan (B), both located in the Pearl River Delta, as locations with good opportunities for teacher training, and Shaoguan (C) and Zhanjiang (D), where access to educational resources is more limited (Guangdong Academy of Education [GAE], 2020). Teacher training programs related to recent education reforms, which attempt to encourage teachers to implement improvement-oriented assessment, are frequently held in these four cities aiming to increase English teachers’ understanding of FA and its implementation.

### Participants

A total of 171 EFL teachers were recruited through purposive sampling. In the qualitative phase, 10 teachers (two male and eight female) were invited to participate in semi-structured interviews (see Table 1 for details). The interviewees represented various teaching stages. Four teachers were from junior high schools, and six were from senior high schools. The participants had different levels of teaching experience (two teachers each had more than 10 years of teaching experience in senior high school). Two teachers were qualified to teach either first-grade or second-grade, thereby reflecting the diversity of teaching abilities. In the quantitative phase, the researchers contacted heads of subject departments to invite teachers to complete questionnaires. A total of 161 teachers completed questionnaires (see Table 2 for details). Participants were teachers from Guangdong province in the four cities mentioned above: A (23.6%), B (28.7%), C (20.4%), and D (27.4%). Most of them (86.6%) held a Bachelor’s degree, and 12.7% had a Master’s degree. The participants in the survey were predominantly female (80.3%). The teachers were drawn in equal proportions from junior and senior high schools. In terms of teaching experience and grades taught, as a result of the recruitment process, the distribution of the samples was relatively even.

**TABLE 1:** Demographic information for the teachers who participated in the interview.

Name	Gender	Years of teaching experience	Education background	Qualifications*	Teaching grade	Teachers’ city
Michelle	Female	24	BA	First-grade teacher	Senior 2	Guangzhou
Ken	Male	8	BA	Second-grade teacher	Junior 2	Guangzhou
Sunny	Female	7	BA	Second-grade teacher	Junior 3	Foshan
Nina	Female	1	MA	Unknown	Senior 1	Foshan
Alice	Female	1	MA	Unknown	Senior 1	Shaoguan
Lily	Female	6	MA	Second-grade teacher	Senior 2	Zhanjiang
Cynthia	Female	6	MA	Second-grade teacher	Senior 2	Guangzhou
Tiffany	Female	3	BA	Second-grade teacher	Junior 1	Shaoguan
Michael	Male	12	MA	First-grade teacher	Senior 3	Shaoguan
Sophia	Female	9	BA	Second-grade teacher	Junior 2	Zhanjiang

*According to Ministry of Education of the People’s Republic of China [MoE] (2015), a teacher’s professional title is bestowed on teachers in the elementary education system, which has five levels: professor, senior teacher, first-grade teacher, second-grade teacher, and third-grade teacher. The MoE criteria cover teachers’ ethics, level of pedagogical content knowledge, and achievements, which can represent teachers’ teaching ability.

**TABLE 2:** Demographic information for the teachers who participated in the survey.

Demographic information	Category	Frequency	Percentage
Gender	Male	31	19.7
	Female	126	80.3
Years of teaching experience	From 1 to 5 years	34	21.7
	From 5 to 10 years	31	19.7
	From 10 to 15 years	45	28.7
	Over 15 years	47	29.9
Educational background	Diploma	1	0.6
	BA	136	86.6
	MA	20	12.7
Qualifications	Second-grade teacher	53	33.8
	First-grade teacher	60	38.2
	Senior teacher	35	22.3
	Others	9	5.7
Teaching grade	Junior 1	28	17.8
	Junior 2	21	13.4
	Junior 3	28	17.8
	Senior 1	15	9.6
	Senior 2	29	18.5
	Senior 3	36	22.9
Teacher’s city	Guangzhou (A)	37	23.6
	Foshan (B)	45	28.7
	Shaoguan (C)	32	20.4
	Zhanjiang (D)	43	27.4

### Instruments

#### Semi-Structured Interview

Semi-structured interviews allow participants to express feelings and provide researchers with rich details of specific experiences by responding to open-ended questions (Cohen et al., 2018). A seven-question interview protocol was developed to achieve the research purpose of this study. This generated an item pool of quantitative data, enabling the researchers to understand Chinese teachers’ FA perceptions and their practice of FA. The interview protocol was cross-checked by the two researchers in order to ensure the clarity and logic of the interview questions. In light of this process, the sequence of the interview questions was changed, and the wording of the last question was revised (see Supplementary Material for the interview protocol).

#### Questionnaire

The purpose of the questionnaire was to investigate the perceptions and practice of FA of English teachers in China and to identify the reasons that may cause a divergence between perceptions and practice. Before participants answered the questions, the research purpose was stated clearly, and they were given assurances regarding the confidentiality of their personal information. Three rating scales were constructed. Each scale had 15 items, with scores ranging from strongly agree (1) to strongly disagree (5). Responses with a lower score indicated a higher degree of agreement with the statement. All the items were designed by drawing on the relevant literature, the interview data, and the TPB. The first rating scale concerned teachers’ beliefs about FA, including its focus, subjects, contents, effects, and the differences between FA and SA. The items were drawn from Heritage’s (2007) identification of the key elements of FA and from Wiliam and Thompson’s (2008) elaboration of the main aspects involved in implementing FA. Consideration was also given to the features of the exam-oriented educational context. The second scale concerned teachers’ FA practice, including feedback, questioning, portfolio assessment, self-assessment, peer assessment, and the formative use of SA. The items were drawn from the strategies identified by Black et al. (2004) and those mentioned in the English curriculum for compulsory education [Ministry of Education of the People’s Republic of China (MoE), 2011] and high school [Ministry of Education of the People’s Republic of China (MoE), 2017].

The third scale was constructed based on Ajzen’s (1985) TPB. With its emphasis on personal attitudes toward behavior, social expectations, and self-efficacy concerns, this theory seems particularly suitable for investigating the possible causes of the unexpected effects of actual FA methods. When this theory is applied to the implementation of FA, specific factors can be identified. Teachers’ beliefs concerning FA, which can be categorized as behavioral attitudes, can impact the effectiveness of FA implementation. According to subjective norms, teachers’ perceptions of social pressures, which can come from important referents such as colleagues, school leaders, and students, also influence teachers’ decisions while implementing FA. This last-mentioned factor is related to perceived behavioral control, indicating that teachers’ perceptions of the ease or difficulty of implementing FA strategies may facilitate or impede their practice. The item pool generated from the interview data contributed to formulating items relating to teachers’ own attitudes, the influence of other stakeholders, and the disadvantages of FA methods. All questionnaires were written in Chinese to avoid language problems because English was the participants’ second language.

To validate the questionnaire, an expert in language testing was invited to check the clarity of the language used, resulting in the adjustment of the instructions and items. The two researchers cross-checked each scale. In addition, a pilot study involving 51 secondary school English teachers was conducted with a Cronbach’s alpha calculated as 0.785, signifying an acceptable level of reliability.

### Data Collection and Analysis

The qualitative research comprised ten semi-structured interviews. The interviews were based on the availability of each interviewee, each taking approximately 30 to 60 min. The interviews were conducted in Chinese, given that teachers would feel more comfortable expressing themselves in their first language. Before the interviews, each participant was informed of its purpose, and confidentiality was assured. Permission to record was obtained from each teacher. During the interview, the researcher took notes and asked follow-up questions to explore the responses, thereby increasing the reliability of the data collection (Cohen et al., 2018). The recorded interviews were transcribed, and the transcripts were sent to the corresponding participants with a request to check for ambiguities. For the data analysis, the interview transcripts were examined, and the data addressing the research questions were coded. Specifically, the transcripts were scanned for the identification of three main themes related to the research questions. Then, the transcripts were read closely in a first round to highlight the keywords. Possible themes began to emerge, for example, “improve students’ learning,” “agents,” and “peer assessment.” After several reviews, similar concepts were combined. Following this process, five themes were derived, featuring teachers’ perceptions. Six themes emerged for the practice of FA, and there were three categories for the reasons that might have caused a gap between conceptual understanding and actual practice. The qualitative data then served as an item pool for the quantitative research.

In the quantitative research phase, each participant spent approximately 10 to 15 min completing the online questionnaires. Four completed questionnaires were discarded because the same option had been selected for all items. Before the participants answered the questions, the research purpose was stated clearly, and they were assured of the confidentiality of their personal information. For the 157 valid questionnaires, the Cronbach’s alphas of the three scales were 0.720, 0.736, and 0.876, respectively, indicating each scale had acceptable or good internal consistency. The first and second research questions were addressed by calculating the frequency, mean score, and standard deviation of each item. Inferential analyses were conducted to explore the relationship between independent variables and causes to answer the third research question. An independent-samples *t*-test was carried out to detect any significant difference in teachers’ attitudes that might be related to gender. One-way ANOVA and *post hoc* analysis within-subjects factors were conducted on the independent variables affecting three or more groups (i.e., years of teaching experience, educational background, professional title, teaching grade, and teaching area).

## Results

### Results of the Interviews

Teachers are important agents in classroom assessment, and their beliefs about FA can influence the effectiveness of its implementation. The interviews demonstrate that participants’ understanding of FA focus and content is consistent with its definition and that teachers can distinguish FA from SA in terms of function:

(1) FA emphasizes the process of students’ learning. It assesses not only students’ academic achievement but also their learning attitude and their classroom performance. It also diagnoses students’ learning needs (Ken).
(2) Literally, FA focuses on the process while SA is about the result. Like the final exam, it just gives a score. We cannot evaluate students’ learning process. But with FA, we can observe their learning process by assessing their presentations (Tiffany).

Teachers mentioned several stakeholders in FA, such as teachers, students, and even parents. It is noteworthy that teachers hold different opinions of the practice of involving parents in FA. Sunny involved parents in giving feedback while Michelle did not:

(1) When students do a presentation, I use my phone to record their performance. Sometimes I send these videos to the students’ parents. This motivates students to perform better because parents would comment on their presentations (Sunny).
(2) My students do not want me to let their parents know too much about their performance because they think their parents meddle too much in their affairs. But, I think it’s a good thing to involve parents in assessing students’ learning (Michelle).

Providing feedback is one of the frequently mentioned methods, and different teachers provide different types of feedback. Ken tended to grade students’ writing assignments with comments while Alice preferred to provide message-related written feedback only considering students’ proficiency:

I usually give a grade and written feedback to students when marking their writing. I feel it is my responsibility to provide comments and not just a score or a grade. I think my students care deeply about my comments (Ken).

I don’t grade my students’ writing. Because their English level is lower than average, they usually get only a few points, which always discourages them. It is meaningless to provide a score. My usual practice is to give comments mainly on the aspects that they need to improve and on those that they have improved (Alice).

The use of questioning to check students’ comprehension of the learning materials is also illustrated. Teachers would design follow-up questions, mainly why and how questions, but they did not consider many higher-order cognitive skills. Regarding the use of a portfolio to assess students, participants noted that they struggled with portfolio assessments because they require substantial time and energy. For example, Cynthia said, “I tried, but it needs time and space to collect and store these materials. It’s not easy to assess the process through them, although I know they are useful.” Among the participants, only one teacher from a junior high school worked with achievement portfolios to assess students’ writing ability. Peer assessment is practiced more often than self-assessment as it can assist students’ language learning and lighten teachers’ burden. For instance, Ken divided students into several groups and asked group members to assess their peers’ writing assignments. Students were provided with rating criteria and were required to write comments. However, some teachers mentioned students’ negative attitudes toward peer assessment. Take Sunny as an example:

My students are unwilling to share and assess work with other students. They like to work alone. I don’t know why. I once asked them to exchange their writing and to proofread each other’s work. They showed a negative attitude. So, I gave up on this method (Sunny).

Almost all the teachers seemed to have a positive attitude toward their use of summative tests. As Sophia explained:

We, including my colleagues, always analyze each test in detail. I do not think that tests are evil. English tests are not only about multiple choice. They are about thinking. Students have to make inferences, interpret, and analyze. We use tests to diagnose students’ difficulties and make specific suggestions (Sophia).

Those responses indicate that teachers have adopted some FA methods in their practice and that they admit to gaps between what the curriculum requires and what happens in the actual classroom. The reasons revealed in the interviews can be summarized as teachers lacking professional knowledge, being influenced by other stakeholders, and the disadvantages of FA methods. The interviews suggested teachers’ uncertainty concerning their FA practice. Some teachers stated honestly that FA was a new term for them. One teacher expressed this as follows:

I am not sure whether my practice constitutes FA. I just based my understanding on its literal meaning and my teaching experience (Sunny).

It seems that Chinese EFL teachers are not sufficiently confident in their practice of FA strategies because they lack the relevant knowledge. For some teachers, the anxiety of having to make students improve their grades can be a major barrier.

(1) I can’t handle extra assessment activities. The existing work already drives me crazy. I can’t balance FA and my teaching. It takes lots of time. I have to improve students’ grades. And the only shortcut is to explain the knowledge points again and again and assign more tests (Nina).
(2) I am really worried about my students’ scores. It’s frustrating when they don’t improve. There is absolutely no time to organize assessment activities like presentations (Tiffany).

The interviews suggest that school leaders, colleagues, and students play a role in the influence exerted by other stakeholders. Some teachers noted that schools did not explicitly demand the implementation of FA. One teacher mentioned their colleagues’ practice of FA:

Well, in my school, there is no such requirement. It all depends on whether teachers want to use it or not. As far as I know, my colleagues seldom or only sometimes get students to assess each other. Personally, I design FA tasks based on whether teaching materials are suitable for carrying out FA strategies. But most of the time, I don’t use them (Sophia).

Other teachers also mentioned that schools do not provide sufficient resources to support FA implementation. Lily’s comment was typical of other teachers’ views:

It is a heavy load to collect students’ learning materials. I wish we were equipped with some digital devices or online platforms. I know some applications give immediate feedback on students’ work (Lily).

There were other influencing factors. The number of students in a class and their English language proficiency could account for the gap between teachers’ perceptions of FA and their actual practice. FA focuses on the learning process of each individual learner. It is not easy for teachers to assess each student in a large class.

(1) You can’t assess everybody. I teach two classes. You can’t imagine what it is like to have over one hundred students. Checking the homework takes most of my time. It would be very difficult to assess and to give individual feedback (Nina).
(2) My students’ English is rather poor. Some can’t write a complete sentence and even don’t know the keywords, so they cannot evaluate their peers’ work (Alice).

Participants indicated that in an exam-oriented context, the disadvantages of FA methods could be a problem. For example, teachers claimed that FA only worked at certain times:

(1) It takes a long time to see the effects of FA. You can’t count on feedback or peer assessment to improve their scores in a short time, although it can bring about some changes to students’ attitudes and to their confidence (Nina).
(2) I think FA methods have a shelf-life. I mean, they can work for a while, but they can’t last for a long time. You have to design different assessment tasks to draw students’ attention. Also, some methods, like portfolio assessment, are time-consuming. Collecting, organizing, and storing students’ work is tedious (Ken).

The results from the interviews suggest that the participating teachers have a superficial understanding of FA and that their daily assessment activities are unsatisfactory. The most significant factors affecting FA implementation are internal, such as teachers’ own perceptions and capacity, and external, such as the influence of others and the disadvantages of FA methods.

### Results of the Questionnaire

Table 3 presents the results of the descriptive statistics for the three scales. The higher the mean score, the stronger the disagreement with the item. For the teachers’ perceptions of FA, the lowest means are reported, in order, for item 6 (“I think there should be more teacher-oriented assessment activities in English language teaching.”) (*M* = 1.19), item 2 (“I think there could be several agents engaged in formative assessment, including teachers, students, and parents.”) (*M* = 1.29), item 1 (“I think the focus of formative assessment should be on improving students’ learning.”) (*M* = 1.31), and item 9 (“I think formative assessment helps to enhance students’ confidence.”) (*M* = 1.31). The highest means are reported, in order, for item 8 (“I think there should be more parent-oriented assessment activities.”) (*M* = 2.96), item 7 (“I think there should be more peer assessment activities.”) (*M* = 1.95) and item 10 (“I think formative assessment can improve students’ English grades.”) (*M* = 1.95). These results show that teachers advocate more teacher-related assessment activities than student- or parent-related ones.

**TABLE 3:** Descriptive statistics of teachers’ perceptions (*N* = 157).

Item	*M*	*SD*	Item	*M*	*SD*	Item	*M*	*SD*
**Perceptions**								
1	1.31	0.62	6	1.19	0.41	11	1.46	0.81
2	1.29	0.59	7	1.95	0.64	12	1.33	0.65
3	1.44	0.79	8	2.96	0.63	13	1.39	0.78
4	1.33	0.62	9	1.31	0.63	14	1.83	0.52
5	1.32	0.59	10	1.95	1.09	15	1.90	0.45
**Practices**								
1	3.81	0.56	6	3.85	0.65	11	3.78	0.64
2	3.68	0.63	7	3.87	0.57	12	3.43	0.63
3	3.09	0.75	8	3.92	0.46	13	2.87	0.71
4	2.25	0.80	9	3.94	0.45	14	1.62	0.99
5	3.82	0.56	10	3.81	0.62	15	1.63	0.99
**Causes**								
1	2.25	0.82	6	2.37	0.88	11	2.06	0.84
2	1.98	0.89	7	2.01	0.85	12	2.15	0.75
3	1.90	0.83	8	2.19	1.01	13	1.89	0.90
4	2.36	1.14	9	2.53	1.19	14	2.20	0.99
5	2.24	1.00	10	2.61	0.97	15	2.34	0.99

The range of standard deviation for teachers’ FA practice is between.45 and.99, indicating that the participants adopt various methods. The lowest means are presented, in sequence, for item 14 (*M* = 1.62), item 15 (*M* = 1.63), and item 4 (*M* = 2.25). Items 14 and 15 concern the formative use of summative tests, and item 4 is “I give a grade or score only for students’ English assignments.” The highest mean scores are shown in order with item 9 (“I collect students’ excellent essays and compile them into a book.”) (*M* = 3.94), followed by item 8 (“I make a portfolio for each student and collect their work to mark their progress.”) (*M* = 3.92), and item 7 (“I design questions based on Bloom’s taxonomy to assess students’ classroom performance.”) (*M* = 3.87).

The lowest means for reasons for the reality of teachers’ FA practice are presented, in order, for item 13 (“Formative assessment, such as constructing rating scales, collecting students essays, etc., increases my daily workload.”) (*M* = 1.89), item 2 (“I have no extra time and energy to conduct formative assessment due to the heavy daily workload.”) (*M* = 1.98), and item 3 (“The pressure of improving students’ summative grades is too great for me to practice formative assessment methods successfully.”) (*M* = 1.90). The highest means are shown, in order, for item 9 (“The students’ English proficiency is too deficient for me to practice formative assessment.”) (*M* = 2.53), item 6 (“Teachers in my school do not practice formative assessment methods, neither do I.”) (*M* = 2.37), and item 4 (“There is no professional training related to formative assessment.”) (*M* = 2.36). Therefore, teachers agree more on the workload and pressure factors than on the influence of other stakeholders.

To gain greater insight into FA implementation, independent-samples *t*-tests and one-way ANOVA tests were adopted to explore whether there were significant differences between teachers regarding the independent variables (i.e., gender, years of teaching experience, educational background, professional title, teaching grade, and teaching area) and their attitudes toward the reasons behind the current situation (i.e., teachers’ attitudes toward FA implementation, influence exerted by other stakeholders, disadvantages of FA). It should be noted that the results of Levene’s tests showed an unequal distribution of participants across different groups, thereby indicating the unsuitability of running one-way ANOVA. Fortunately, the data transformation to the base 10 logarithm for the variable for each subject satisfies the assumption of homogeneity of variance. The results are presented in Tables 4, 5.

**TABLE 4:** Independent-sample *t*-test of gender (*N* = 157).

	Group							
	Male (*n* = 31)		Female (*n* = 126)					
Item	*M*	*SD*	*M*	*SD*	*t*	Sig.	95% of CI	η^2^
Teachers’ own attitude	1.20	0.62	1.23	0.50	0.64	0.52	(−0.14, 0.28)	0.00
Influence of other stakeholders	2.53	1.09	2.51	1.04	0.11	0.92	(1.39, 0.44)	0.00
Disadvantages of FA	0.92	0.47	0.86	0.43	0.72	0.48	(−0.11, 0.24)	0.00

**TABLE 5:** Results of One-way ANOVA.

Levene’s Test for Equality of Variances						
**Independent variable**	**Dependent variable**	***F***	**Sig.**	***F***	**Sig.**	***η^2^***
Teaching experience	Teachers’ own attitude	0.61	0.61	1.93	0.13	0.04
	Influence of other stakeholders	0.18	0.91	1.83	0.14	0.03
	Disadvantages of FA methods	1.71	0.17	0.90	0.44	0.02
Educational background	Teachers’ own attitude	1.72	0.19	0.48	0.62	0.01
	Influence by other stakeholders	1.25	0.27	3.93	0.02	0.05
	Disadvantages of FA methods	3.14	0.08	0.65	0.52	0.01
Professional title	Teachers’ own attitude	1.26	0.29	0.18	0.91	0.00
	Influence of other stakeholders	0.81	0.49	0.09	0.96	0.00
	Disadvantages of FA methods	1.17	0.33	0.66	0.58	0.01
Teaching grade	Teachers’ own attitude	0.85	0.36	0.83	0.36	0.01
	Influence of other stakeholders	0.91	0.34	3.01	0.09	0.02
	Disadvantages of FA methods	2.05	0.15	2.92	0.09	0.02
Cities	Teachers’ own attitude	1.69	0.17	3.50	0.02	0.06
	Influence of other stakeholders	1.05	0.37	9.17	0.00	0.15
	Disadvantages of FA methods	3.18	0.03	0.50	0.68	0.01

The independent-samples *t*-test analysis of gender showed a non-significant difference between reasons for FA implementation: teachers’ own attitude (*p* = 0.52 > 0.05, *η^2^* = 0.00), influence of other stakeholders (*p* = 0.92 > 0.05, *η^2^* = 0.00), and disadvantages of FA methods (*p* = 0.48 > 0.05, *η^2^* = 0.00). In terms of independent variables of teaching experience, no statistical difference was found between teachers’ own attitude [*F*(3, 153) = 1.93, *p* = 0.13 > 0.05, *η^2^* = 0.04], influence of other stakeholders [*F*(3, 153) = 1.83, *p* = 0.14 > 0.05, *η^2^* = 0.03], and disadvantages of FA methods [*F*(3, 153) = 0.90, *p* = 0.44 > 0.05, *η^2^* = 0.02]. The same results were found for the independent variables, professional title and teaching grade. The data suggest that the independent variables of gender, years of teaching experience, professional title, and teaching grade did not influence teachers’ responses to items relating to the three reasons. For teachers with different educational backgrounds, a statistically significant difference was found between teachers with a Bachelor’s degree and teachers holding a Master’s degree with regard to the influence of other stakeholders [*F*(3, 153) = 3.93, *p* = 0.02 < 0.05, *η^2^* = 0.05].

In terms of the variable of teachers’ own attitudes, the Fisher’s least significant differences *post hoc* test results, presented in Table 6, show that the mean scores for teachers from city B were significantly higher than those for teachers from cities C (*p* = 0.02 < 0.05) and D (*p* = 0.00 < 0.05). Regarding the variable of influence of other stakeholders, *post hoc* tests also show that the mean scores for teachers from city A were significantly higher than those for teachers from cities B (*p* = 0.01 < 0.05) and D (*p* = 0.02 < 0.05).

**TABLE 6:** *Post hoc* tests on the differences between cities.

Variable	Teachers’ city		Mean difference	Sig.	95% of CI
**Teachers’ own attitude**					
	Foshan (B)	Shaoguan (C)	0.27	0.02	(0.04, 0.51)
	Foshan (B)	Zhanjiang (D)	0.33	0.00	(0.12, 0.55)
**Influence of other stakeholders**					
	Guangzhou (A)	Foshan (B)	0.66	0.01	(0.19, 1.12)
	Guangzhou (A)	Zhanjiang (D)	0.52	0.02	(0.09, 0.96)
	Foshan (B)	Shaoguan (C)	1.01	0.00	(0.56, 1.45)
	Foshan (B)	Zhanjiang (D)	0.87	0.00	(0.46, 1.28)

In short, the results indicate that the state of the current classroom practice of FA is not positive. Specifically, the data reveal that teachers’ deep understanding of FA principles has not been fully developed. The results of the survey of classroom practice demonstrate that the extent of the implementation of FA methods in the exam-oriented educational context is limited. FA practice is closely linked to SA practice, and the formative use of summative tests is the most acceptable strategy. The possible reasons for the current situation can be categorized as three factors, teachers’ own perceptions toward FA theories, the influence of the important referents, and the limitations of FA methods. In addition, teachers’ attitudes toward these factors differ according to their educational background and the region.

## Discussion

This study attempts to contribute to our understanding of the features and underlying causes of implementing FA within an exam-oriented education system.

### Reality of Formative Assessment Practice

With regard to RQ 1, the consistent findings reported in the qualitative and quantitative data show that, in general, EFL teachers in Chinese secondary schools know the FA construct and its effects but are profoundly lacking in understanding. These findings are consistent with a case study conducted by Wang (2017), which found that Chinese secondary teachers have implicit knowledge of FA. Teachers’ superficial understanding of FA and its methods can influence their decisions on using the FA approach (Carless, 2011). Regarding FA strategies, it seems that peer assessment is welcomed by teachers as students can generate constructive feedback, especially in a class with a large number of students (Ballantyne et al., 2002). Previous studies highlighted the role of parents and advocated collaboration between teachers and parents (Sach, 2012). However, the findings of this study yield the opposite result, namely that teachers do not have a positive attitude toward parental involvement. A possible reason could be the Chinese context, in which parents play a major role in students’ learning, with a tendency toward excessive interference in students’ lives leading to adverse impacts. Parents’ excessive concern regarding students’ achievement may shape teaching and learning (Linn, 1993).

The second research question addresses the FA practice of Chinese EFL teachers in public secondary schools. The findings suggest that teachers adopt diverse methods with different degrees of frequency. Data from the interviews affirm the role of feedback without grades or scores, confirming Lee and Wiliam’s (2005) study, which asserted that students would pay more attention to teachers’ feedback when it is given without grades or scores. However, the findings from the questionnaire did not show widespread use. This may be attributable to the process being time-consuming. In the context of Chinese secondary education, EFL teachers are in charge of one or two classes, each class having more than forty students, indicating a huge workload. In such circumstances, it is understandable that the use of grades only is widespread, although this is criticized for its negative impact on learning (Wiliam, 2017). Heavy workloads can cause teachers’ resistance to FA methods (Hopfenbeck et al., 2015). For example, the need to document students’ assessments requires extra work and time.

Secondary education in China is exam-oriented, suggesting that teachers and students place extreme emphasis on grades and rankings. It is not surprising that teachers make full use of summative tests to assess whether students’ learning practice is efficient. Previous studies have reported the necessity to establish coherent FA and SA systems to encourage teachers to carry out varied assessment practices to facilitate students’ learning (Broadfoot and Black, 2004). The findings from this study support the notion that test follow-up activities can be used as a formative strategy in an exam-dominated context (Carless, 2011). In line with previous studies (Stiggins, 1992), the findings show that questioning cultivates students’ critical thinking but that teachers fail to frame quality questions. As Volante and Beckett (2011) showed, teachers tend to use old questioning methods rather than using Anderson and Krathwohl’s (2001) taxonomy. Self-assessment is often considered as a complement to peer assessment (Black et al., 2004). However, contrary to the findings of these studies, the present study shows that self-assessment is not used as widely as peer assessment. The current study tentatively interprets this to be because EFL teachers in Chinese public secondary schools do not understand how to conduct self-assessment by students. This situation results from the lack of training in designing valid questionnaires or scales to evaluate students’ learning process (Heritage, 2010).

Although the findings of the current investigation are not positive, they have value in that they may draw the attention of EFL teachers in China to the formative use of summative tests in their particular context. They need to know that FA strategies are closely related to their daily classroom practice. The present research shows that the exam-oriented educational context has a significant impact on how assessment is carried out in the classroom. The strategy of the formative use of summative tests is one of the situated FA strategies. Black and Wiliam (2006) stressed that the principal purpose of FA is to support learning and to enable teachers to develop ways of making formative use of summative tests. The authors proposed that teachers could allow students to mark their own test papers in their peer groups, thereby freeing up time for discussion of particularly difficult questions. As reflected in the findings of the present study, teachers could make full use of exercises and tests to instruct students to reflect on areas where their learning seemed insufficient. It is also suggested that teachers could train students preparing for examinations to generate and to answer their own questions. The involvement of students in the learning process could make them feel that they could benefit from summative tests.

### Factors in Formative Assessment Implementation

Concerning the third research question, this study explains the findings in terms of TPB (Ajzen, 1985). The findings of this study show that teachers lack deep FA-related knowledge. This is the result of insufficient teacher training and a lack of self-directed professional development, which can adversely impact teachers’ confidence in putting FA into practice (Volante and Beckett, 2011). The findings also suggest that teachers endure a heavy daily workload and that they are under tremendous pressure to improve students’ grades. In the Chinese context, it is reasonable to note that the general high-stakes pressure has a great impact on teachers’ attitudes toward FA implementation (Cheng and Qi, 2006). This is also consistent with the findings of Brown et al. (2011), which show that teachers value accountability for improved grades and that they are sensitive to adverse consequences due to incorrect interpretations of students’ performance in examinations. The fact that teachers’ perceptions of this reason for poor FA implementation has statistical significance by city may result from the rapid, and sometimes uneven, development of the economy. This situation should be addressed by providing additional professional teacher training programs.

The influence exerted by other stakeholders is a second factor of importance. The attitudes of school leaders, colleagues, and students exert a specific effect on teachers’ willingness to implement FA. An individual’s intention is likely to be influenced by the attitudes of important others (Ajzen, 1985). Participants also mentioned that their colleagues do not take FA seriously because of their school leaders’ indifferent attitude toward FA. As stated above, the number of students in a Chinese EFL class is too large for the teacher to oversee the learning process of each individual learner. Class size may therefore influence a teacher’s intention to conduct FA. It is impossible to practice FA effectively without students’ cooperation and involvement (Heritage, 2007). The results suggest that peer assessment, for example, is not practiced efficiently because some students refuse to assess their peers’ work. This is partially in line with the findings of Sach’s (2012) study. As Klimenko and Sleptsova (2015) noted, the possible cause of this refusal is that students do not know exactly how to assess others’ work and have not truly understood the function of peer assessment activities. This, in turn, could be attributed to teachers’ unclear classroom instructions. Statistically, significant differences are found in Chinese EFL teachers’ perceptions of this situation by city and by educational background. This is probably due to the relatively good educational resources available in the developed cities of Guangzhou and Foshan and the less favorable situation in Shaoguan and Zhanjiang. The postgraduate education of some teachers is another factor to be considered.

The disadvantages of the various FA methods constitute another noteworthy factor. The ANOVA results indicate no statistically significant difference between the teachers’ biographical information (i.e., gender, years of teaching experience, educational background, professional title, teaching grade, and teaching area) and their view of this factor. Therefore, it may be inferred that most participants acknowledge the disadvantages of some FA methods. The disadvantages mentioned in this study suggest that the extra work involved in applying FA methods is onerous and that some of the methods are difficult to implement in actual classroom settings. This finding corresponds to similar findings of studies reporting that teachers are deterred by the complicated work involved in methods such as keeping portfolios (Fox, 2014; Lyon et al., 2021).

In line with the framework established in the literature review, this study uncovers the mechanism of FA implementation in the exam-dominated context from the social psychological perspective. The reality of FA practice was affected by the teachers’ own perceptions, the influence of other stakeholders, and by the disadvantages of FA methods, all of which correspond to the TPB. Teachers’ negative beliefs about the efficacy of FA methods would influence their willingness to perform FA. This suggests that teacher trainers should be cautious about the interpretation of FA theories and principles and that they should build teachers’ confidence in the practice of FA. Teachers’ perceptions of social pressure are also a major factor. Their decisions to transform FA methods into practice are influenced by their social network. Policy-makers should be more aware of the impact of related stakeholders and should target them to explain and promote FA practice. The typical features of schooling in China, such as teachers’ workload, time constraints, and class size, raise teachers’ concerns regarding the disadvantages of FA methods. These factors seem to exert a negative influence on teachers’ willingness to perform FA.

## Conclusion

The present study explored FA practice in Chinese public secondary schools from the teachers’ perspective, examining factors that may influence its implementation under the framework of the TPB. The findings show that Chinese EFL teachers are not well informed about FA theory. The study also shows that the teachers prefer to use those FA methods that are easy to control and involve less work. From the TPB perspective, teachers’ beliefs about attitudes toward FA, along with the influence of other stakeholders and the disadvantages of FA methods, were found to be the root causes of this situation.

The study enriched our understanding of FA in a secondary educational context, in which all stakeholders are under heavy examinations pressure. It should be noted that the implementation of the FA mechanism in some circumstances is complex. It is also susceptible to the influence of the local educational context. The use of the TPB to probe the implementation of FA in an exam-oriented context contributes to the existing knowledge of FA from two aspects. First, it has the potential to gain a complete and structural understanding of EFL teachers’ decisions regarding FA and the underlying reasons behind their attitudes and practices. Previous studies have tended to focus on the effectiveness of FA strategies. The investigation of the factors related to teachers’ attitudes, subjective norms, and perceived behavioral control has extended our knowledge of how teachers’ attitudes, the influence of other stakeholders, and the disadvantages of the FA methods could have a role in shaping the current FA situation. Second, it provides a process-oriented perspective examining the FA mechanism. According to the TPB, a person’s behavioral responses to a trigger can best be understood as the outcome of the interaction between attitudes, subjective norms, and behavioral control (Ajzen, 1985, 1991). In this study, TPB provides a framework to describe how these three factors work together to shape the teachers’ FA implementation intentions. Teachers’ beliefs about FA would influence their attitude toward FA, their perceptions of social pressure, and their attitude toward their FA insufficiency. The combination of these factors would influence teachers’ decisions on their actual teaching behavior. The pedagogical implications are that EFL teachers need to be aware of the benefits of FA. They should be encouraged to enhance their willingness to know more about FA and to adapt FA methods to students’ learning styles and to daily classroom assessment practice. Teacher training programs should include instruction in FA to improve teachers’ knowledge and practical skills. School leaders need to make an effort to minimize the creation of an environment in which teachers focus exclusively on students’ grades rather than their learning process. Teachers’ collaborative learning should be encouraged with a view to nurturing strong relationships between colleagues. Schools should build professional learning communities to develop FA practice. Policy-makers need to change the exam-oriented nature of school practice and create favorable conditions for integrating FA into the assessment system. The *Gaokao* and *Zhongkao* should be the only gate-keeping exams for learners.

This study has some limitations. Semi-structured interviews may not provide sufficient data to frame a questionnaire, and it may not be possible to generalize the results to other educational contexts or to a society that is quite different from Chinese context. Moreover, drawing on the case of Guangdong province, this study addressed the reality of FA practice and its underlying reasons only from the point of view of teachers. Other evidence such as data from classroom observation could be considered in the future investigation of EFL teachers’ daily assessment practice. In addition, future researchers could explore the perceptions of other stakeholders, for example, students. Finally, further comparative studies should be conducted of FA practice in countries with exam-oriented education systems and in countries with less exam-dominated education systems.

## Data Availability Statement

The raw data supporting the conclusions of this article will be made available by the authors, without undue reservation.

## Ethics Statement

The studies involving human participants were reviewed and approved by The Center for Language Cognition and Assessment, School of Foreign Studies, South China Normal University, Guangzhou, China. Written informed consent for participation was not required for this study in accordance with the national legislation and the institutional requirements.

## Author Contributions

JZ collected and analyzed the data and wrote the initial draft of the manuscript. LH conceived the study and revised the manuscript substantially. Both authors contributed to the article and approved the submitted version.

## Conflict of Interest

The authors declare that the research was conducted in the absence of any commercial or financial relationships that could be construed as a potential conflict of interest.

## Publisher’s Note

All claims expressed in this article are solely those of the authors and do not necessarily represent those of their affiliated organizations, or those of the publisher, the editors and the reviewers. Any product that may be evaluated in this article, or claim that may be made by its manufacturer, is not guaranteed or endorsed by the publisher.
