# Supplementary material for: Understanding Formative Assessment Practice in the EFL Exam-Oriented Context: An Application of the Theory of Planned Behavior
Source: Front Psychol. 2021 Dec 9;12:774159. doi: 10.3389/fpsyg.2021.774159 (PMC8695487; doi:10.3389/fpsyg.2021.774159)
Supplement: Supplementary file 1 [file Data_Sheet_1.pdf]

Supplementary Material

1 Interview Protocol

(1) What is your understanding of formative assessment?

(2) Have you adopted formative assessment methods? If yes, how do you implement them?

(3) Have you come across difficulties when practicing these methods? If yes, how do you deal with them?

(4) Do you think formative assessment methods improve your students' academic achievement?

(5) What do you think formative assessment is supposed to be?

(6) What is the gap between your actual teaching practice of formative assessment and your ideal one?

(7) What do you think are the reasons for the gap?

2 Questionnaire

Part I *Demographic information*

In this part, please provide the following information by writing down your response in the brackets.

(1) Gender (   )

A. Male B. Female

(2) Years of teaching experience (   )

A. From 1 to 5 years B. From 5 to 10 years C. From 10 to 15 years D. Over 15 years

(3) Educational background (   )

A. Diploma B. BA C. MA D. PhD E.________

(4) Qualifications (   )

A. Second-grade teacher B. First-grade teacher C. Senior teacher D.______

(5) Teaching grade (   )

A. Junior 1 B. Junior 2 C. Junior 3 D. Senior 1 E. Senior 2 F. Senior 3 G._______

(6) The city you currently work in (   )

A. Guangzhou B. Foshan C. Shaoguan D. Zhanjiang

Supplementary Material

Part II ***Teachers' perceptions of formative assessment***

In this part, we provide the following statements about what you think of formative assessment. Please give your answers honestly to guarantee the success of the survey.

We would like you to tell us how much you agree or disagree with the statements by ticking (√) a number from 1 to 5.

My perceptions of formative assessment: 1 strongly **agree**, 2 agree, 3 I am not sure, 4 disagree, 5 strongly **disagree**	
(1) I think the focus of formative assessment should be on improving students' learning.	1-----2-----3-----4-----5
(2) I think there could be several agents engaged in formative assessment, including teachers, students, and parents.	1-----2-----3-----4-----5
(3) I think formative assessment in English language teaching can evaluate students' thinking capacity.	1-----2-----3-----4-----5
(4) I think formative assessment in English language teaching can directly influence students' learning.	1-----2-----3-----4-----5
(5) I think formative assessment in English language teaching can assess all aspects of learning.	1-----2-----3-----4-----5
(6) I think there should be more teacher-oriented assessment activities in English language teaching.	1-----2-----3-----4-----5
(7) I think there should be more peer assessment activities.	1-----2-----3-----4-----5
(8) I think there should be more parent-oriented assessment activities.	1-----2-----3-----4-----5
(9) I think formative assessment helps to enhance students' confidence.	1-----2-----3-----4-----5
(10) I think formative assessment can improve students' English grades.	1-----2-----3-----4-----5
(11) I think formative assessment can help me identify students' English learning needs and adjust my teaching plan.	1-----2-----3-----4-----5
(12) I think formative assessment can adjust students' learning attitudes toward English language learning.	1-----2-----3-----4-----5
(13) I think formative assessment in English language teaching can facilitate student interaction in the classroom.	1-----2-----3-----4-----5
(14) I think I can accurately tell the differences between formative assessment and summative assessment.	1-----2-----3-----4-----5
(15) I think formative assessment in English language teaching can compensate for the disadvantages of summative assessment.	1-----2-----3-----4-----5

Part III ***Teaching practices of formative assessment***

In this part, we provide the following statements about your teaching practice of formative assessment. Please give your answers honestly to guarantee the success of the survey.

We would like you to tell us how much you agree or disagree with the statements by ticking (√) a number from 1 to 5.

My practice of formative assessment: 1 strongly **agree**, 2 agree, 3 I am not sure, 4 disagree, 5 strongly **disagree**	
(1) I write comments only on students' English assignments.	1-----2-----3-----4-----5
(2) I write comments, and I give a grade or score for students' English assignments.	1-----2-----3-----4-----5
(3) I give oral feedback on students' presentations.	1-----2-----3-----4-----5
(4) I give a grade or score only for students' English assignments.	1-----2-----3-----4-----5
(5) I talk to students individually about their recent English learning performance.	1-----2-----3-----4-----5
(6) I design quality questions to assess students' thinking skills.	1-----2-----3-----4-----5
(7) I design questions based on Bloom's taxonomy to assess students' classroom performance.	1-----2-----3-----4-----5
(8) I make a portfolio for each student and collect their work to mark their progress.	1-----2-----3-----4-----5
(9) I collect students' excellent essays and compile them into a book.	1-----2-----3-----4-----5
(10) I use electronic tools or platforms to store students' work.	1-----2-----3-----4-----5
(11) I provide criteria for students to conduct self-assessment.	1-----2-----3-----4-----5
(12) I provide criteria for students to conduct peer assessment.	1-----2-----3-----4-----5
(13) I allow students to work in groups on a project, and I assess them as a group.	1-----2-----3-----4-----5
(14) I use students' grades in summative tests to understand their recent learning performance.	1-----2-----3-----4-----5
(15) I analyze students' performance in summative tests to give specific feedback and instructions.	1-----2-----3-----4-----5

Part IV ***Teachers' perceptions of the reasons for the reality of formative assessment implementation***

In this part, we provide the following statements about your perception of the reasons for the reality of formative assessment implementation. Please give your answers honestly to guarantee the success of the survey.

We would like you to tell us how much you agree or disagree with the statements by ticking (√) a number from 1 to 5.

My practice of formative assessment: 1 strongly **agree**, 2 agree, 3 I am not sure, 4 disagree, 5 strongly **disagree**	
(1) I do not fully understand the notion of formative assessment.	1-----2-----3-----4-----5
(2) I have no extra time and energy to conduct formative assessment due to the heavy daily workload.	1-----2-----3-----4-----5
(3) The pressure of improving students' summative grades is too great for me to practice formative assessment methods successfully.	1-----2-----3-----4-----5
(4) There is no professional training related to formative assessment.	1-----2-----3-----4-----5
(5) Schools do not explicitly require teachers to practice formative assessment.	1-----2-----3-----4-----5
(6) Teachers in my school do not practice formative assessment methods, neither do I.	1-----2-----3-----4-----5
(7) The number of students in a class is too large to practice formative assessment.	1-----2-----3-----4-----5
(8) The range of students' English proficiency is too large to practice formative assessment.	1-----2-----3-----4-----5
(9) The students' English proficiency is too deficient for me to practice formative assessment.	1-----2-----3-----4-----5
(10) Students are not willing to engage in formative assessment methods (e.g., peer assessment).	1-----2-----3-----4-----5
(11) Students lose interest in formative assessment methods making it difficult for me to keep using the same methods.	1-----2-----3-----4-----5
(12) The procedures of most formative assessment methods are too complicated to implement.	1-----2-----3-----4-----5
(13) Formative assessment, such as constructing rating scales, collecting students' essays, etc., increases my daily workload.	1-----2-----3-----4-----5
(14) My school lacks the necessary facilities to make the practice of formative assessment convenient.	1-----2-----3-----4-----5
(15) Formative assessment cannot improve students' academic achievement in a short time.	1-----2-----3-----4-----5
